# Data supporting the improvement of forecasting and control of electricity consumption in hotels

**DOI:** 10.1016/j.dib.2019.104147

**Published:** 2019-06-12

**Authors:** José Cabello Eras, Vladimir Sousa Santos, Alexis Sagastume Gutierrez, Carlo Vandecasteele

**Affiliations:** a Universidad de la Costa, Calle 50 No 55-66, PBX 336 22 00, Calle 58 # 55 e 66, Atlántico, Barranquilla, Colombia; b Department of Chemical Engineering, University of Leuven, de Croylaan 46, B-3001 Heverlee, Belgium

**Keywords:** Energy management, Buildings energy efficiency, Electricity consumption

## Abstract

Improving and managing the electricity efficiency in hotel facilities is essential to reduce the hotel operation costs and its environmental impacts. The data presented shows the evolution of the electricity consumption and management between 2013 and 2015 in two hotel facilities in Cuba (one beach hotel and one city hotel). The data additionally includes the daily measures used to develop control tools for an energy management system. The data presented in the article relates to the research study: *Tools to improve forecasting and control of the electricity consumption in hotels* Cabello et al., 2016, and it corresponds to the energy audits developed in one beach hotel (Hotel A) and one city hotel (Hotel B) in Cuba.

**Table:** Specifications table

Subject area	Energy
More specific subject area	Energy Management
Type of data	Table, graph, figure (raw data related to each data files is provided)
How data was acquired	Own measurements and calculations, based on: • Hotel electric meters • Hotel records • Measurements with an IP power meter of four channels (that includes a multi-channel power meter Pilot (model PMAC211)) • Measurements with a power quality and energy analyzer Fluke 435, series 6
Data format	Raw, filtered, analyzed.
Data source location	Cienfuegos, Cuba
Data accessibility	Data are with this article
Related research article	Tools to improve forecasting and control of the electricity consumption in hotels. https://doi.org/10.1016/j.jclepro.2016.07.192[1].

**Table:** 

**Value of the data** • The dataset can be used as starting point to forecast and manage the electricity consumption in Cuban hotels. • The dataset can be used, as benchmark to compare the behavior of hotel facilities in tropical areas, for specialists in the energy management of buildings. • The dataset can be used as a reference to benchmark the energy performance of hotels in tropical areas. • The data can be used in courses of energy efficiency and management in buildings, as a detailed example of the implementation and evolution of the energy efficiency and management measures in hotel facilities. • The data serve as a guidance to target specific areas and equipment during a measurement campaign in hotel facilities. • The data shows the evolution of the electricity consumption of two hotel facilities during the implementation of energy efficiency measures within an energy management system.

## 1 Data

The data includes the occupied rooms per day (ORD), the outdoor temperature and the electricity consumption on daily and on monthly basis. It additionally includes the parameters calculated and used to develop the electricity management of the hotel facilities (i.e. room degree-day (RDD), energy performance indicators (EnPI), energy baselines (EnB) and control graphics), to forecast and control the electricity consumption in hotels A and B, by highlighting their main sources of inefficiencies. Originally, the dataset [1] considered data from 2011 to 2012 to develop the EnBs and EnPIs used. This data was updated up to 2015 to show the evolution of the electricity management system and the control tools over time.

**Fig. 1:**
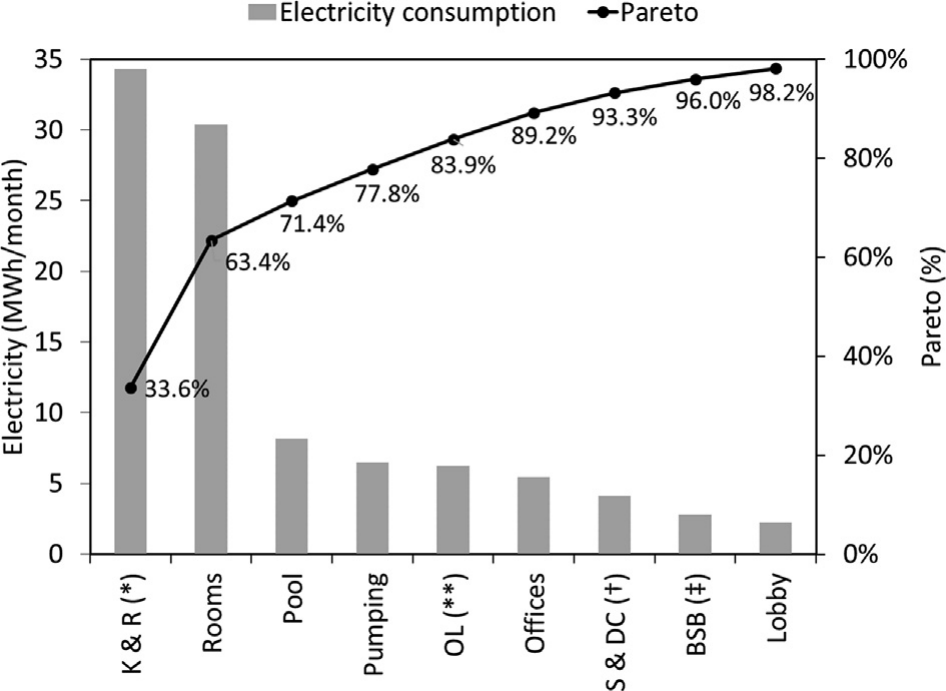
Pareto of the electricity consumption by areas (Hotel A), * Kitchen and restaurant, ** Outdoor lighting, † Shops and dance club, ‡ Beach snack bar.

**Fig. 2:**
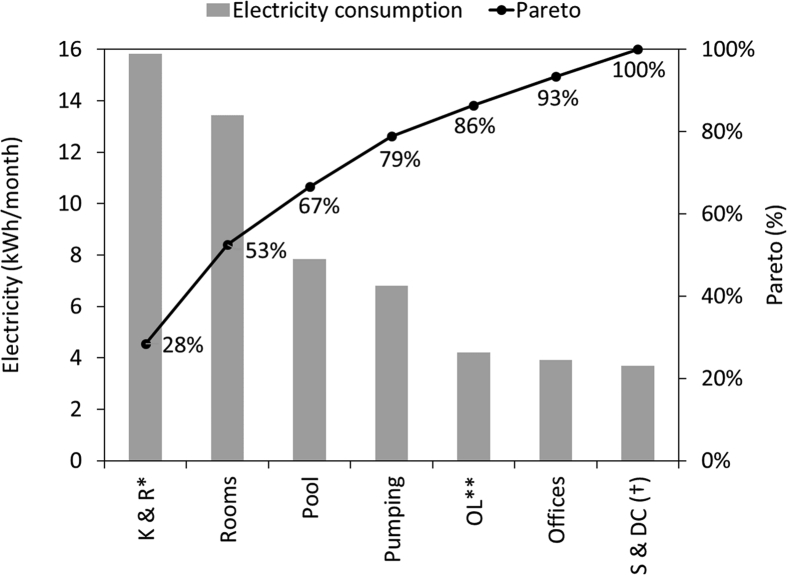
Pareto of the electricity consumption by areas (Hotel B). * Kitchen and restaurant, ** Outdoor lighting, † Shops and dance club.

**Fig. 3:**
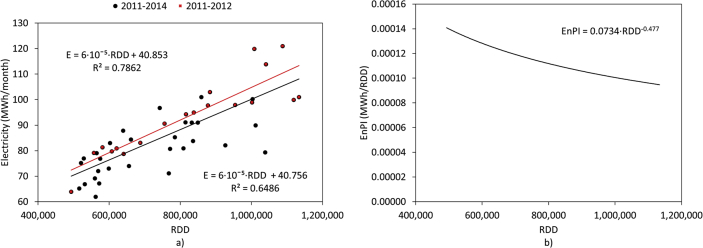
Monthly EnPl and control graphics (Hotel A).

**Fig. 4:**
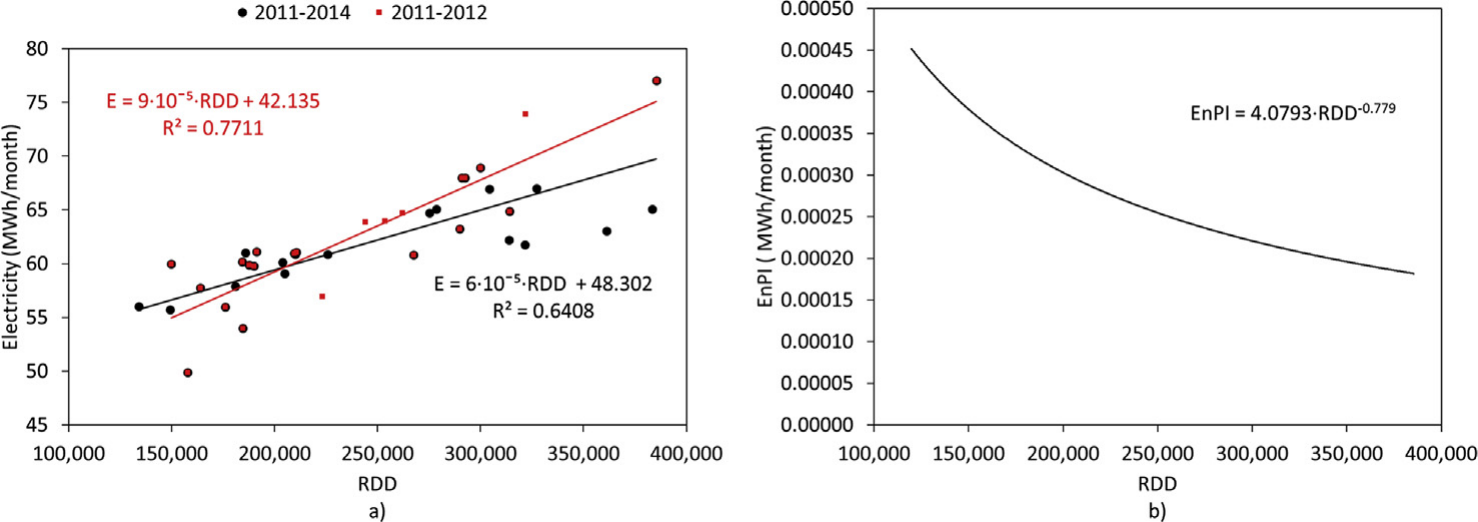
Monthly EnPl and control graphics (Hotel B).

**Fig. 5:**
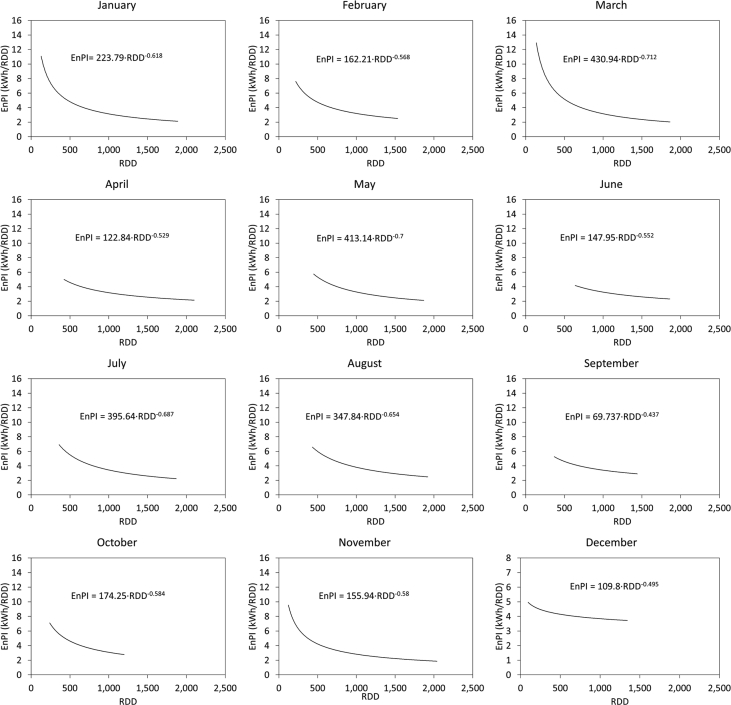
Daily control graphs (Hotel A).

**Fig. 6:**
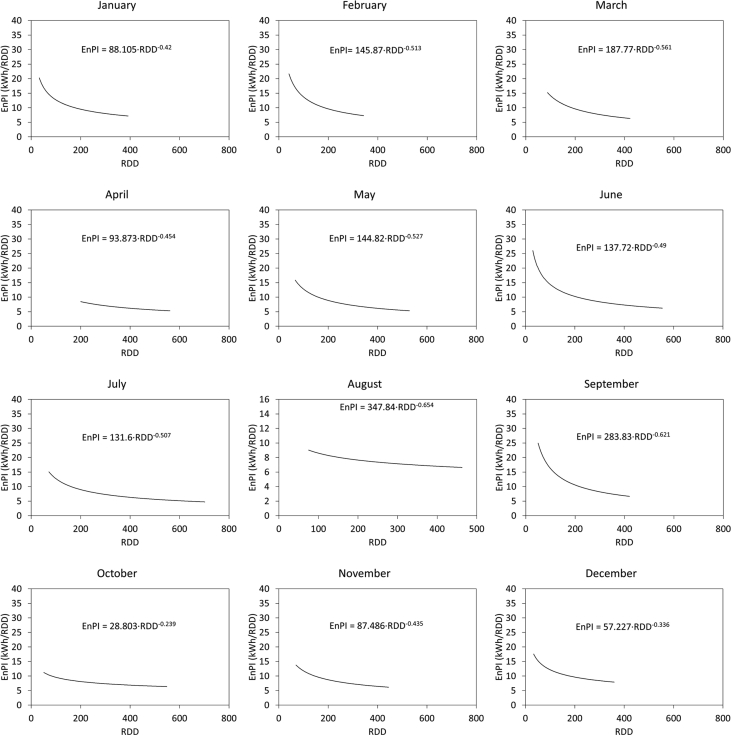
Daily control graphs. Hotel B.

**Fig. 7:**
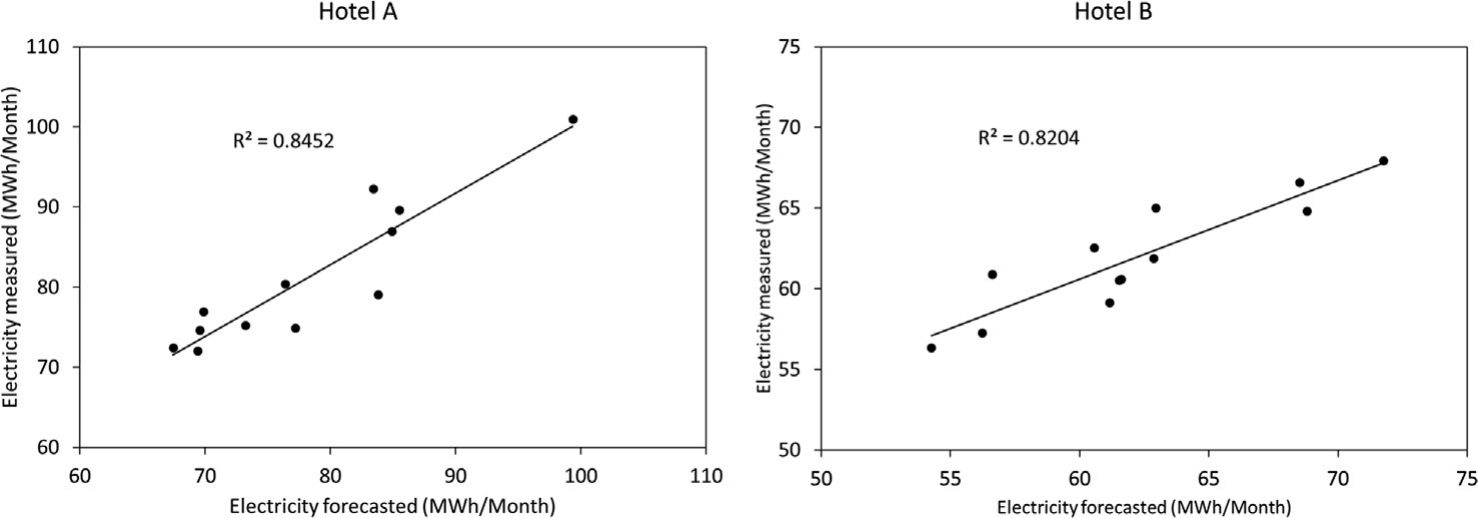
Scatter analysis of the measured and forecasted electricity consumption on monthly basis (2015).

Fig. 1 and Fig. 2 show the average electricity consumption of the different areas for hotels A (Fig. 1) and B (Fig. 2). Moreover, Fig. 3 and Fig. 4 show the electricity consumption between 2011 and 2014 for both hotels, and additionally show the Energy Performance Indicators (EnPI) developed for hotels A and B. Furthermore, the daily control graphs, developed for each month based on the daily electricity consumption measured in each hotel are shown in Fig. 5 and Fig. 6. Finally, Fig. 7 shows a scatter analysis between the measured and the forecasted electricity consumption in hotels A and B. In addition, Table 1 shows the monthly electricity consumption measured between January 2011 and December 2014 and the reference parameters calculated based on the measurements. Moreover, Table 2 shows the monthly electricity consumption measured during 2015 and the reference parameters calculated based on the measurements. The data used to develop Fig. 1, Fig. 2, Fig. 3, Fig. 4, Fig. 5, Fig. 6 is available in the article.

**Table 1:** Monthly data and reference parameters calculated (January 2011–December 2014).

Month	Hotel A				Hotel B			
	Electricity $\left(\frac{MWh}{Month}\right)$	ORD	CDD	RDD	Electricity $\left(\frac{MWh}{Month}\right)$	ORD	CDD	RDD
Jan-2011	83.134	5,798	119	687,256	55.990	1,294	136	176,015
Feb-2011	78.767	5,247	122	640,055	49.887	1,292	122	157,605
Mar-2011	90.999	5,544	150	831,600	61.064	1,404	150	210,600
Apr-2011	97.793	3,970	221	877,370	68.912	1,357	221	299,897
May-2011	96.758	2,860	260	742,170	63.896	940	260	243,930
Jun-2011	102.99	3088	286	883,168	60.154	644	286	184,184
Jul-2011	94.993	2,773	302	837,446	68.000	963	302	290,826
Aug-2011	119.924	3,267	309	1,007,870	67.996	948	309	292,458
Sep-2011	79.172	1,970	283	556,525	59.959	530	283	149,725
Oct-2011	81.313	2,220	262	580,530	64.738	1,002	262	262,023
Nov-2011	79.788	3,262	186	606,732	63.982	1,363	186	253,518
Dec-2011	63.989	2,987	165	492,855	61.124	1,158	165	191,070
Jan-2012	85.291	5,788	136	784,274	59.868	1,385	135	187,532
Feb-2012	90.692	6,190	122	755,087	57.778	1,344	122	163,948
Mar-2012	97.983	6,358	150	953,700	60.949	1,397	150	209,550
Apr-2012	101.046	5,129	221	1,133,509	63.225	1,311	221	289,731
May-2012	98.996	3,860	260	1,001,670	64.899	1,210	260	313,995
Jun-2012	99.954	3,910	286	1,118,260	72.670	891	286	254,826
Jul-2012	113.93	3,445	302	1,040,390	77.058	1,276	302	385,352
Aug-2012	120.976	3,524	309	1,087,154	73.937	1,043	309	321,766
Sep-2012	94.317	2,886	283	815,295	59.759	672	283	189,840
Oct-2012	83.836	3,196	262	835,754	60.804	1,023	262	267,515
Nov-2012	79.371	5,584	186	1,038,624	56.976	1,200	186	223,200
Dec-2012	80.994	3,759	165	620,235	54.006	1,118	165	184,470
Jan-2013	87.871	5,323	120	638,760	60.876	1,304	161	209,944
Feb-2013	84.436	5,461	121	660,781	55.705	1,224	122	149,310
Mar-2013	91.177	5,430	150	814,500	60.141	1,387	147	203,889
Apr-2013	100.201	4,601	218	1,003,018	65.085	1,260	221	278,460
May-2013	83.055	2,321	260	602,300	64.705	1,046	263	275,098
Jun-2013	80.766	2,733	282	770,706	59.097	716	286	204,776
Jul-2013	76.918	1,901	302	574,102	66.983	1,087	301	327,187
Aug-2013	76.998	1,727	306	528,462	66.933	986	309	304,181
Sep-2013	75.214	1,843	283	520,648	56.008	479	280	134,120
Oct-2013	79.034	2,137	264	564,168	60.866	863	262	225,675
Nov-2013	100.998	4,616	186	858,576	64.963	1,256	189	237,384
Dec-2013	72.011	3,512	162	568,944	57.891	1,096	165	180,840
Jan-2014	82.121	5,716	162	925,992	61.043	1,397	133	185,801
Feb-2014	74.005	5,459	120	655,048	45.068	1,272	125	159,000
Mar-2014	80.961	5,428	149	808,732	53.098	1,478	150	221,700
Apr-2014	89.975	4,599	220	1,011,721	62.228	1,401	224	313,764
May-2014	73.011	2,319	258	598,232	61.781	1,237	260	321,550
Jun-2014	71.149	2,731	281	767,335	49.351	907	284	257,511
Jul-2014	67.171	1,899	301	571,518	65.058	1,278	300	383,319
Aug-2014	66.937	1,725	308	531,217	63.062	1,177	307	361,256
Sep-2014	65.191	1,841	280	515,404	37.897	670	179	119,882
Oct-2014	69.139	2,135	262	559,299	57.073	1,246	260	323,890
Nov-2014	91.005	4,614	184	848,926	56.142	1,447	191	276,325
Dec-2014	61.987	3,510	160	561,557	48.971	1,387	163	226,081

**Table 2:** Measured and forecasted values of the reference parameters and the electricity consumption on monthly basis (2015).

Month	Hotel A					Hotel B				
	ORD	CDD	RDD	ECM* $\left(\frac{MWh}{month}\right)$	ECF** $\left(\frac{MWh}{month}\right)$	ORD	CDD	RDD	ECM $\left(\frac{MWh}{month}\right)$	ECF $\left(\frac{MWh}{month}\right)$
Jan	5,323	120	638,760	83.802	79.082	1,304	161	209,944	56.625	60.899
Feb	5,461	121	660,781	76.383	80.403	1,224	122	149,310	56.221	57.262
Mar	5,430	150	814,500	85.512	89.626	1,387	147	203,889	61.528	60.535
Apr	4,601	218	1,003,018	99.338	100.937	1,260	221	278,460	62.950	65.010
May	2,321	260	602,300	69.875	76.964	1,046	263	275,098	68.790	64.808
Jun	2,733	282	770,706	84.897	86.998	716	286	204,776	61.598	60.589
Jul	1,901	302	574,102	73.197	75.202	1,087	301	327,187	71.760	67.933
Aug	1,727	306	528,462	67.432	72.464	986	309	304,181	68.512	66.582
Sep	1,843	283	520,648	69.408	72.050	479	280	134,120	54.262	56.349
Oct	2,137	264	564,168	69.565	74.606	863	262	225,675	62.866	61.868
Nov	4,616	186	858,576	83.424	92.271	1,256	189	237,384	60.559	62.545
Dec	3,512	162	568,944	77.189	74.893	1,096	165	180,840	61.152	59.152

*ECM – electricity consumption measured; **ECF – electricity consumption forecasted

## 2 Experimental design, materials, and methods

The monthly electricity consumption was taken from the electric bills of each hotel, while the daily consumption was taken from the mandatorily measures take every day at 7 a.m. in the electric meter of every hotel by the maintenance staff, as requested by the Cuban Ministry of Tourism to keep track of the electricity consumption of the tourist sector. Other data (e.g. occupied rooms per days, occupied rooms per month, etc.), was taken from each hotel records.

The electricity consumption per areas used to identify the main electricity uses (as depicted in Fig. 1, Fig. 2), was measured with two IP power meter of four channels to measure 4 areas simultaneously with each one. Additionally, a power quality and energy analyzer Fluke 435 series 6 was used. Moreover, the electricity consumption of the hotel was directly taken from the hotel electric meter. The areas on each hotel were measured during one month, Fig. 1, Fig. 2 shows the average values.

Similar to Ganguly [4], the climatic year (i.e. a continuous 12-month period with a complete annual cycle), developed using 30 years of daily temperature data [5], available in the Weather Underground Database [6], was used to forecast the daily outdoor temperature.

The Room Degree Day (RDD) is calculated as:

(1) RDD=ORD.CDD

where CDD stands for Cooling Degree Day, which is calculated as [7]:

(2) $$\text{CDD}=\sum \left(\emptyset_{0}-\emptyset_{b}\right)$$

where $\emptyset_{0}\;$is the outdoor temperature, and $\emptyset_{b}$ is the reference temperature (maximum outdoor temperature at which no cooling is required to maintain the thermal comfort in a building). The reference temperature must be individually determined for each building [7].

The monthly electricity consumption was forecasted during 2013 and 2014 using the correlation between the electricity consumption and the RDD for hotels A (equation (3)) and B (equation (4)), originally with data from 2011 to 2012:

(3) $$\text{E}=6.47\cdot 10^{-5}\cdot \text{RDD}+40.262$$

(4) $$\text{E}=8.69\cdot 10^{-5}\cdot \text{RDD}+41.856$$

Fig. 3, Fig. 4 show the monthly EnBs used during 2013 and 2014, based on data from 2011 to 2012, which was updated in 2015 using data from 2013 to 2014. Additionally, Fig. 3, Fig. 4 show the EnPI control graphs for hotels A and B on monthly basis. Moreover, Fig. 5, Fig. 6 show the daily control graphs used for each month in hotels A and B. Finally, Fig. 7 shows a scatter analysis between the measured and forecasted electricity consumption in hotels A and B.

Equations (3), (4)) were updated including data from 2013 to 2014 to forecast and manage the consumption during 2015:

(5) $$\text{E}=6\cdot 10^{-5}\cdot \text{RDD}+40.756$$

(6) $$\text{E}=6\cdot 10^{-5}\cdot \text{RDD}+48.302$$

Table 1 shows the monthly electricity consumption and the reference parameters between January 2011 and December 2014, while Table 2 shows the same data measured and calculated during 2015, when the updated control tools, depicted in figures Fig. 3, Fig. 4, Fig. 5, Fig. 6 were implemented. Additionally, Table 2 includes the electricity consumption forecasted with the updated EnPI and the monthly reference parameters during 2015.
